# Perioperative outcomes of robot-assisted laparoscopic radical prostatectomy (RALRP) and LRP in patients with prostate cancer based on risk groups

**DOI:** 10.1080/2090598X.2020.1750865

**Published:** 2020-04-15

**Authors:** Napat Amornratananont, Kun Sirisopana, Suchin Worawichawong, Panas Chalermsanyakorn, Premsant Sangkum, Suthep Pacharatakul, Charoen Leenanupunth, Wisoot Kongchareonsombat

**Affiliations:** a Division of Urology, Department of Surgery, Faculty of Medicine, Ramathibodi Hospital, Mahidol University, Bangkok, Thailand; b Division of Clinical Pathology, Department of Pathology, Faculty of Medicine, Ramathibodi Hospital, Mahidol University, Bangkok, Thailand; c Division of Urology, Department of Surgery, Police Hospital, Bangkok, Thailand

**Keywords:** Prostate cancer, radical prostatectomy, laparoscopy, robot-assisted laparoscopic, risk stratification, perioperative outcomes

## Abstract

**Objective:**

To compare the perioperative and pathological outcomes between robot-assisted laparoscopic radical prostatectomy (RALRP) and LRP based on the patient’s risk.

**Patients and methods:**

The medical records of 588 patients with prostate cancer who underwent RP, using minimally invasive surgery (MIS) techniques (240 LRP and 348 RALRP) by a single surgeon during January 2008 to June 2018 at the Ramathibodi Hospital, were retrospectively reviewed. The patient’s risk was classified according to the National Comprehensive Cancer Network (NCCN) Guideline, 2018. The demographic, perioperative, and pathological data of patients were collected. The differences in perioperative and pathological outcomes between LRP and RALRP in each risk classification were assessed using chi-square, Fisher’s exact tests and logistic regression, as appropriate.

**Results:**

In terms of positive margins, RALRP had significant advantages in high-risk patients when compared to LRP (adjusted odds ratio 0.46, 95% confidence interval 0.26–0.84), while there were no differences in the low- and intermediate-risk patients. Overall, the patients who underwent RALRP had significant advantages over those who underwent LRP in terms of operative time, estimate blood loss, and blood transfusion rate. While, adjacent organ injury rate and length of hospital stay were similar for both techniques in all subgroup analyses.

**Conclusion:**

MIS techniques appear to be safe, especially RALRP, which has significantly better perioperative outcomes in all subgroups of patient risk classification, and in the high-risk patient group it seems to have better pathological outcomes when compared to LRP.

**Abbreviations:**

EBL: estimated blood loss; LOS: length of hospital stay; PSM: positive surgical margin; (L)(O)(RAL)RP: (laparoscopic) (open) (robot-assisted laparoscopic) radical prostatectomy; MIS: minimally invasive surgery

## Introduction

Prostate cancer is the fourth most common cancer affecting Thai men [1] and is a major health concern worldwide, being the second most common neoplasm and sixth cause of cancer-related deaths in the world [2]. Today, robot-assisted laparoscopic radical prostatectomy (RALRP) is the mainstay for the local control of the disease. However, the procedure is associated with significant morbidity and decline in quality of life because of the sequelae of the surgical procedure (urinary incontinence and erectile dysfunction) [3]. In the past decade, especially in Asia, the RP technique has shown a significant trend toward minimally invasive surgery (MIS) techniques [4–6]. Many studies show that MIS techniques (e.g. RALRP and LRP) have some advantages over a traditional open RP (ORP) technique in terms of the operative field and perioperative outcomes (operative time, estimated blood loss [EBL], blood transfusion rate, adjacent organ injury, oncological outcomes, and functional outcomes) [4–7]. It is evident that MIS techniques are safe and have significantly better perioperative outcomes with comparable pathological/oncological outcomes compared to ORP.

There are plenty of study data from high-volume centres that have reported on LRP and RALRP as MIS techniques worldwide. A meta-analysis [8] showed that RALRP, with its advantages of few postoperative complications and well-preserved urinary continence and erectile function, was an effective, and safe option for prostate cancer [8,9]. In Thailand, the cost of treatment with RALRP and LRP differs [10], so the total cost is a factor that determines the choice of treatment in shared doctor–patient decisions. Advocates claim greater precision in dissection and suturing, and accelerated attainment of surgical competency of the more costly RALRP over the standard LRP. There are few studies that compare the two techniques (LRP and RALRP) in depth in terms of subgroup analysis, especially in the high-risk patient group to reveal the advantages of one over the other.

## Patients and methods

### Population and surgical techniques

This study was a single-centre single-surgeon retrospective observational study. In total, 716 patients with prostate cancer treated with RP by a single surgeon at the Ramathibodi Hospital, Mahidol University, Bangkok, Thailand, between January 2008 and June 2018, were reviewed. There were 588 patients who underwent MIS techniques (240 RALRP and 348 LRP) and 128 patients who underwent ORP. All patients who underwent MIS techniques were included in the study analysis. The LRPs were performed in an extraperitoneal fashion and the RALRPs were performed using the da Vinci® Surgical system Si (Intuitive Surgical Inc., Sunnyvale, CA, USA). The operation was selected for each patient depending on the shared decision of the patient and the doctor.

### Baseline characteristic and preoperative parameter

All patients who underwent MIS techniques (RALRP and LRP) were categorised by risk stratification using the National Comprehensive Cancer Network (NCCN) Guideline, version 4 (2018), for subgroup analysis. Patients’ demographic data including age, weight, body mass index (BMI), serum PSA level, clinical stage, and biopsy Gleason score, were collected from the patient medical records and laboratory reports.

### Grouping

All patients who underwent MIS techniques (RALRP and LRP) were divided into two groups depending on the surgical approach. The patient risk was classified according to the NCCN Guidelines, version 4 published in 2018, for the subgroup analysis. Briefly, a patient with a PSA level <10 ng/mL and Gleason score ≤6 was categorised in the low-risk group; a patient with a PSA level of 10–20 ng/mL and Gleason score 7 was categorised in the intermediate-risk group; and a patient with a PSA level of >20 ng/mL and Gleason score ≥8 was categorised in the high-risk group.

### Outcome of interest

The perioperative outcomes, including: operative time, EBL, blood transfusion, adjacent organ injury (bowel injury, unattended bladder injury, or vessel injury), and length of hospital stay (LOS), were retrospectively collected from medical records. The pathological outcomes, e.g. margin status, were retrieved from the pathological reports, which had been examined by experienced pathologists in the Division of Clinical Pathology. Other parameters from the pathological reports, e.g. prostatic weight, biopsy Gleason score, and pathological stage were also collected.

### Statistical analysis

For descriptive statistics, means ± standard deviations (SDs), medians with interquartile ranges (IQRs) and proportions, were used to describe and summarise the collected study data as appropriate. The independent *t*-test, Wilcoxon rank-sum test or Kruskal–Wallis test were used to determine the differences of continuous parameters between surgical approaches or amongst the risk groups. For categorical data, chi-squared or Fisher’s exact test, as appropriate, was used to test the differences amongst groups. Univariate and multivariate logistic regressions were further used to identify and explore the factors associated with the study outcomes. The analysis was performed using STATA Statistical Software: release 14.0 (StataCorp., College Station, TX, USA) and a *P* < 0.05 was considered statistically significant.

## Result

### Patients and prostate cancer stages and grades

The patients’ demographic data, prostate cancer stages and grades are presented in Tables 1 and 2. The data were statistically different in terms of median age between LRP and RALRP only in the high-risk patient subgroup, at 70 (67–73) and 67 (63–72) years, respectively (*P* = 0.019). The median body weight in the high-risk RALRP patient subgroup was statistically significantly higher than that in the high-risk LRP subgroup, at 67 (59.5–75.2) and 64.2 (57.8–71.0) kg, respectively (*P* = 0.028). The median height and median BMI were not different between LRP and RALRP amongst the high-, intermediate-, and low-risk patient groups. Based on reported medical history (hypertension, diabetes mellitus, or dyslipidaemia) there was no difference between LRP and RALRP in all subgroups. The median PSA level in the LRP group was significantly higher than that in the RALRP group only in the high-risk patient group, at 32.3 (21.2–48.8) and 24 (12.4–44.1) ng/mL, respectively (*P* = 0.041). There were no differences in terms of preoperative PSA levels between LRP and RALRP patients in other groups (intermediate- and low-risk groups). The biopsy Gleason score in RALRP patients was significantly higher than that in LRP patients in the high-risk patient group, at 8 and 7, respectively (*P* = 0.025), but there were no differences between LRP and RALRP patients in the other groups.

**Table 1.** Patients’ demographics by surgical approach and risk group.

Variable	LRP	RALRP	*P* by approach	*P* by risk level
No. of patients, *n* (%)	240 (40.8)	348 (59.2)	–	–
High risk	81 (33.8)	141 (40.5)	–	0.013*
Intermediate risk	97 (40.4)	151 (43.4)	–	
Low risk	62 (25.8)	56 (16.1)	–	
Median (IQR):				
Age, years	68 (63–72)	67 (63–72)	0.394	–
High risk	70 (67–73)	67 (63–72)	0.019*	0.023*
Intermediate risk	67 (63–72)	68 (63–71)	0.771	
Low risk	66.5 (62–71)	67 (61–70)	0.766	
Body weight, kg	66.4 (59–72.9)	68 (62–74.6)	0.029*	–
High risk	64.2 (57.8–71)	67 (59.5–75.2)	0.028*	0.176
Intermediate risk	67.8 (60–74.2)	68.1 (63–74)	0.305	
Low risk	66.8 (59.6–74)	66.8 (61.9–73.8)	0.624	
Height, cm	165 (162–169)	165 (162–170)	0.396	–
High risk	165 (161–168)	165 (161–168)	0.591	0.028†
Intermediate risk	165.5 (162–170)	166 (162–170)	0.437	
Low risk	166 (162–170)	166 (162–170)	0.670	
BMI, kg/m^2^	24.2 (21.9–26.5)	24.5 (22.5–26.8)	0.126	–
High risk	24 (21.4–26.2)	24.4 (22.3–27.3)	0.112	0.659
Intermediate risk	24.2 (22.3–26.6)	24.8 (23–26.7)	0.417	
Low risk	24.5 (22.7–26.4)	24.3 (22.3–26.3)	0.989	
Preoperative PSA level, ng/mL	10.6 (7.4–21.2)	11.8 (8–20)	0.141	–
High risk	32.3 (21.2–48.8)	24 (12.4–44.1)	0.041*	<0.001*
Intermediate risk	10.6 (7.8–12.3)	11.5 (7.7–14.2)	0.195	
Low risk	6.9 (5.5–8.4)	7.9 (6.5–8.9)	0.038*	
*N* (%):				
Hypertension	149 (62.1)	211 (60.6)	0.731	–
High risk	55 (67.9)	83 (58.9)	0.198	0.696
Intermediate risk	55 (56.7)	92 (60.9)	0.512	
Low risk	39 (62.9)	36 (64.3)	1.000	
Diabetes mellitus	59 (24.6)	89 (25.6)	0.847	–
High risk	26 (32.1)	42 (29.8)	0.763	0.047*
Intermediate risk	21 (21.7)	36 (23.8)	0.758	
Low risk	12 (19.4)	11 (19.6)	1.000	
Dyslipidaemia	88 (36.7)	134 (38.5)	0.666	–
High risk	28 (34.6)	56 (39.7)	0.475	0.836
Intermediate risk	32 (33.0)	59 (39.1)	0.348	
Low risk	28 (45.2)	19 (33.9)	0.260	

*Statistical significance at *P* < 0.05.

Comparison of the proportions between LRP and RALRP or amongst risk classification groups by Fisher’s exact test.

Comparison of continuous data between LRP and RALRP by the independent *t*-test or Wilcoxon rank-sum test.

Comparison of continuous data amongst risk classification groups by Kruskal–Wallis test.

**Table 2.** Clinical and pathological prostate cancer stages and grades by surgical approaches and risk group.

Cancer stage and grade		Clinical stage				Pathological stage			
		LRP	RALRP	*P* by approach	*P* by risk level	LRP	RALRP	*P* by approach	*P* by risk level
Overall, *n* (%) or *n/N*	T1a	4/5	1/5	0.036*					
	T1b	0/3	3/3						
	T1 c	207 (41.7)	289 (58.3)						
	T2	–	–						
	T3a	7 (26.9)	19 (73.1)						
	T3b	14 (29.2)	34 (70.8)						
	T4	–	–						
High risk, *n* (%) or *n/N*	T1a	–	–		0.459	–	–	0.249	
	T1b	0/1	1/1			–	–		
	T1 c	60 (39.0)	94 (61.0)			–	–		
	T2a	–	–			3/6	3/6		
	T2b	–	–			2/2	0/2		
	T2 c	–	–			25 (32.1)	53 (68.0)		
	T3a	6 (26.1)	17 (73.9)			23 (39.7)	35 (60.3)		
	T3b	12 (30.0)	28 (70.0)			27 (35.5)	49 (64.5)		
	T4	–	–			1/1	0/1		
Intermediate risk, *n* (%) or *n/N*	T1a	0/1	1/1		0.349	–	–	0.284	
	T1b	0/2	2/2			–	–		
	T1 c	93 (39.7)	141 (60.3)			–	–		
	T2	–	–			–	–		
	T2a	–	–			14 (58.3)	10 (41.7)		
	T2b	–	–			3 (50.0)	3 (50.0)		
	T2 c	–	–			42 (35.0)	78 (65.0)		
	T3a	–	–			23 (38.3)	37 (61.7)		
	T3b	1/7	6/7			15 (41.7)	21 (58.3)		
	T4	–	–			–	–		
Low risk, *n* (%) or *n/N*	T1a	4/4	0/4		0.143	–	–	0.464	
	T1b	–	–			–	–		
	T1 c	54 (50.0)	54 (50.0)			–	–		
	T2	–	–			–	–		
	T2a	–	–			18 (62.1)	11 (37.9)		
	T2b	–	–			4/5	1/5		
	T2 c	–	–			30 (47.6)	33 (52.4)		
	T3a	1/3	2/3			7 (43.8)	9 (56.3)		
	T3b	1/1	0/1			2/4	2/4		
	T4	–	–			–	–		
Gleason score, mean (IQR)		7 (6–7)	7 (6–8)	<0.001*	<0.001*	7 (7–7)	7 (7–8)	0.027*	<0.001*
High risk		7 (7–8)	8 (7–9)	0.025		7 (7–9)	8 (7–9)	0.530	
Intermediate risk		6 (6–7)	7 (6–7)	0.282		7 (7–7)	7 (7–7)	0.354	
Low risk		6 (6–6)	6 (6–6)	0.879		6.5 (6–7)	7 (6–7)	0.733	
Prostate volume, mL, median (IQR)									
Overall						38.3 (29.3–50.0)	37.7 (30.0–48.0)	0.933	0.749
High risk						40.1 (32.5–49.4)	36.0 (28.8–44.8)	0.088	
Intermediate risk						37.9 (29.2–47.3)	39.2 (31.2–50.5)	0.481	
Low risk						35.2 (27.3–51.0)	39.7 (30.0–50.0)	0.450	

*Statistical significance at *P* < 0.05.

Comparison of the proportions between LRP and RALRP or amongst risk classification groups by Fisher’s exact test.

Comparison of continuous data between LRP and RALRP by the independent *t*-test or Wilcoxon rank-sum test

Comparison of continuous data amongst risk classification group by Kruskal–Wallis test.

Gleason score at clinical stage was from biopsy.

Gleason score at pathological stage was from prostate tissues removed at radical prostatectomy.

The pathological stage was significantly different in each risk subgroup (*P* = 0.001). The overall median biopsy Gleason score was significantly higher in RALRP than in LRP. The prostate volume (measured by specimen weight) was not significantly different amongst all subgroups for LRP and RALRP.

### Perioperative and pathological outcomes

The perioperative outcomes showed that operative time was significantly shorter for RALRP than for LRP, at 190 and 210 min, respectively (*P* = 0.015). Patients who underwent RALRP had a significantly lower EBL and blood transfusion rate than those who underwent LRP in all subgroup analyses. There was no statistical difference between the MIS techniques, LRP and RALRP, in all subgroup analyses for adjacent organ injury and LOS (Table 3).

**Table 3.** Perioperative and pathological outcomes by surgical approach and risk groups.

Outcomes	LRP (*n* = 240)	RALRP (*n* = 348)	*P* by approach	*P* by risk level
Perioperative outcomes				
Median (IQR):				
Operation time, min	210 (170–260)	190 (165–240)	0.015*	–
High risk	205 (165–240)	190 (160–230)	0.370	0.241
Intermediate risk	210 (165–270)	190 (165–240)	0.073	
Low risk	225 (170–270)	195 (170–242.5)	0.159	
EBL, mL	500 (300–800)	300 (200–500)	<0.001*	–
High risk	400 (300–800)	300 (200–500)	<0.001*	0.052
Intermediate risk	400 (250–800)	300 (200–500)	0.018*	
Low risk	500 (300–1000)	400 (250–500)	0.001*	
*N* (%):				
Blood transfusion	53 (23.1)	20 (5.8)	<0.001*	–
High risk	19 (23.5)	10 (7.1)	0.001*	0.986
Intermediate risk	21 (22.6)	9 (6.0)	<0.001*	
Low risk	13 (23.6)	1 (1.8)	0.001*	
Adjacent organ injury	6 (2.6)	2 (0.6)	0.066	–
High risk	2 (2.5)	0 (0.0)	0.135	0.424
Intermediate risk	2 (2.1)	1 (0.7)	0.562	
Low risk	2 (3.6)	1 (1.8)	1.000	
Hospitalisation time, days, median (IQR)	6 (5–8)	5 (4–8)	0.121	–
High risk	6 (5–8)	6 (4–8)	0.475	0.174
Intermediate risk	6 (5–8)	6 (5–8)	0.858	
Low risk	5 (5–8)	5 (4–6)	<0.001*	
Pathological outcome				
PSM, *n* (%)	96 (41.2)	126 (36.3)	0.258	<0.001*
High risk	48 (59.3)	58 (41.1)	0.012*	
Intermediate risk	35 (36.5)	54 (35.8)	1.000	
Low risk	13 (23.2)	14 (25.5)	0.828	

Abbreviations: EBL: estimate blood loss; PSM: positive surgical margin.

*Statistical significance at *P* < 0.05.

Comparison of proportions between methods or risk groups by Fisher’s exact test.

Comparison of continuous outcomes between methods by Independent t-test or Wilcoxon rank-sum test. Comparison of continuous outcomes among risk groups by Kruskal-Wallis test.

For the pathological outcomes, the positive surgical margin (PSM) rate was only significantly different in the high-risk patient group, at 41.1% and 59.3% for RALRP and LRP, respectively (*P* = 0.012). After adjusting for other variables (Table 4) using multivariate logistic regression, the results still showed that the high-risk patients who underwent RALRP had a significantly lower risk of PSMs when compared to those who underwent LRP (adjusted odds ratio [OR] 0.46, 95% CI 0.26–0.84). The multivariate analysis also showed that the high-risk patients with higher PSA levels and higher biopsy Gleason scores were associated with higher risk of PSMs (OR 1.01, 95% CI 1.00–1.02; and OR 1.54, 95% CI 1.16–2.07, respectively).

**Table 4.** Factors associated with marginal outcomes in the high-risk group (*n* = 222).

		*N*	PSM, *n* (%)	Univariate analysis OR (95% CI)	*P*	Multivariate analysis OR (95% CI)	*P*
MIS technique	LRP	81	48 (59.3)	Reference	0.010*	Reference	0.011
	RALRP	141	58 (41.1)	0.48 (0.27–0.84)		0.46 (0.26–0.84)	
Preoperative PSA level (ng/mL)		219	104 (47.49)	1.01 (1.00–1.02)	0.010*	1.01 (1.00–1.02)	0.021
Biopsy Gleason score		217	103 (47.47)	0.88 (0.68–1.14)	0.330		
Pathological Gleason score		221	106 (47.96)	1.54 (1.17–2.05)	0.002*	1.54 (1.16–2.07)	0.003
Prostate volume (mL)		209	100 (47.85)	1.00 (0.99–1.00)	0.597		
Age (years)		222	106 (47.75)	0.97 (0.93–1.02)	0.224		
Body weight (kg)		222	106 (47.75)	1.00 (0.97–1.03)	0.768		
Height (cm)		221	105 (47.51)	1.00 (0.95–1.05)	0.989		
BMI (kg/m^2^)		221	105 (47.51)	1.02 (0.95–1.09)	0.649		
Hypertension	No	84	43 (51.19)	Reference	0.423		
	Yes	138	63 (45.65)	0.80 (0.46–1.38)			
Diabetes	No	154	76 (49.35)	Reference	0.472		
	Yes	68	30 (44.12)	0.81 (0.46–1.44)			
Dyslipidaemia	No	138	67 (48.55)	Reference	0.759		
	Yes	84	39 (46.43)	0.92 (0.53–1.58)			

*Statistical significance at *P* < 0.05.

Significant variables from univariate analysis were included in multivariate logistic regression model.

Backward elimination methods were used to identify the significant factor in multivariate logistic regression analysis.

## Discussion

Two recent studies [11,12] demonstrated that RALRP is at least equivalent to ORP or LRP in terms of PSM rates and suggested that RALRP provides certain advantages, especially regarding decreased risk of adverse events. Although patients with high-risk prostate cancer have increased incidence of biochemical recurrence and requirement for secondary therapy, there are many treatment options available for this patient group, e.g. RP, radiation in combination with androgen-deprivation therapy, or observation amongst others, with a tendency to select RP. A previous study showed that the pathological and oncological outcomes in patients with high-risk prostate cancer were similar amongst RALRP, LRP and ORP [13]. In our present study, only in the high-risk patient group was RALRP superior to LRP for PSM rates. The RALRP allows for more precise instrument movement when dissecting and improved operative field visualisation compared with LRP. According to our present results, if the patient is classified as high risk, RALRP is preferred as it results in better oncological outcomes. To confirm this difference in PSM rates, more prospective, randomised controlled trials are needed.

At the Ramathibodi Hospital, RP is the standard treatment for localised and locally advanced prostate cancer. Since 2007, the surgical trend toward LRP for prostate cancer is obvious [14–16], with LRP being used as the standard approach. In 2013, RALRP was introduced at the Ramathibodi Hospital. In many studies in the past, RALRP has been shown to be more advantageous than LRP [17] in terms of EBL, operative time, blood transfusion rate, LOS, and adjacent organ injury rate. According to previous studies [11,18], there are ample data from high-volume centres reporting that RALRP has the advantages of fewer postoperative complications compared with LRP. Similar to our present study, the operative time, EBL, and blood transfusion rate were significantly lower for RALRP than for LRP. When comparing the LOS, in our present study we found no statistically significant difference between LRP and RALRP (6 and 5 days, respectively). In fact, in our experience, the LOS can be affected by many other factors such as patient preference, socioeconomic status, and disease complications, and these may explain why there was no difference between RALRP and LRP in our present study. In regards to the adjacent organ injury rate, there was no statistically significant difference between LRP and RALRP in all subgroup analyses, which is probably explained by the fact that both RALRP and LRP are MIS techniques with good visualisation of the surrounding anatomy.

Our present study has some limitations. With the retrospective study design, both known and unknown factors between LRP and RALRP could not be controlled for, such as medication given prior to the surgery. Furthermore, the improvement of surgical performance overtime by the surgeon could be a confounding factor when evaluating the outcomes of the procedure, although different techniques adopted by the single surgeon might reduce the bias from surgical performance amongst surgeons.

## Conclusion

The MIS techniques of LRP and RALRP appear to be promising techniques for the treatment of organ-confined prostate cancer and some locally advanced prostate cancers. Comparing LRP and RALRP, RALRP seems to have a shorter operative time, lesser EBL, and lower blood transfusion rate than LRP, but there was no difference in the adjacent organ injury rate and LOS. In regard to the pathological outcome, it was found that RALRP was superior to LRP for the PSM rate, but only in the high-risk patient group. As RALRP was more beneficial in terms of pathological outcome than LRP in the high-risk patient group in the present study, we therefore highly recommend RALRP for the treatment of prostate cancer in high-risk patients.
